# Sortase-click strategy for defined protein conjugation on a heptavalent cyclodextrin scaffold

**DOI:** 10.1371/journal.pone.0217369

**Published:** 2019-05-24

**Authors:** Shikha Singh, Kanchan Gupta, Shagun Shukla, Srinivasa-Gopalan Sampathkumar, Rajendra P. Roy

**Affiliations:** National Institute of Immunology, Delhi, India; National Cancer Institute at Frederick, UNITED STATES

## Abstract

Multivalent proteins or protein dendrimers are useful for clinical and biotechnological applications. However, assembly of chemically defined protein dendrimers is a challenging endeavor. In the past, majority of protein dendrimers have been developed on branched lysine scaffolds and are usually limited to a valency of two to four. The naturally occurring cyclodextrin (CD) scaffold composed of 6–8 glucose units offers the possibility of expanding the valency. Here we have adapted a chemoenzymatic-click strategy for displaying heptavalent peptides and large proteins on the β-cyclodextrin (β-CD) scaffold. We demonstrate that recombinant proteins (engineered with a LPXTG pentapeptide motif at the carboxy terminus), labeled with an alkyne moiety by sortase-mediated ligation, can be easily clicked on to the azide-derivatized β-cyclodextrin through the Huisgen cycloaddition reaction yielding a well-defined heptavalent display of proteins.

## Introduction

Cyclodextrins (CD) are natural cyclic oligomers composed of six (α), seven (β) or eight (γ) D-glucose units linked by α- 1, 4- glycosidic bonds **[1]**. Cyclodextrins have a torus architecture with a hydrophobic cavity that allows inclusion of spatially compatible molecules **[2–4].** The noncovalent inclusion complexes of cyclodextrin have attracted a wide variety of applications for stabilization of drugs **[5],** solubilization of peptides and proteins, protein folding **[6]** etc. Besides, CD scaffold also comprises one of the most easily accessible scaffolds for multivalent display of ligands. The hydroxyl groups on CD surface are endowed with differential reactivity and are amenable to suitable modifications with azide, alkynes, esters, sugars and other functionalities for further elaboration with a variety of macromolecules derivatized with compatible orthogonal groups **[7–11]**.

In recent years, CD has been found as an attractive scaffold for covalent display of peptide ligands **[12–14]**.The synthesis of β-CD, symmetrically substituted with phenylalanine and cysteine residues, was reported by Ashton et al almost two decades ago **[15]**. This work exploited the selective derivatization of primary hydroxyl groups to an amine for carbodiimide-mediated coupling of Boc-amino acids. Subsequently, bimodal conjugates of δ-aminolevulinic acid and β-CD with an average substitution of three was prepared through ester linkages **[9]**. A mono-functionalized conjugate of oxytocin, a divalent conjugate of a 24-mer peptide derived from bZIP transcription factor, and a heptavalent conjugate of a 12-mer peptide respectively, with β-CD has been reported **[12–14]**. The aforementioned examples clearly demonstrate the feasibility of using β-CD as a scaffold for covalent display of peptide ligands. However, it is pertinent to note that a symmetrical substitution leading to heptavalent occupancy of peptide ligands beyond a 12-mer peptide on β-CD has not been reported thus far.

The assembly of well-defined dendrimers comprising longer peptide sequences or large proteins is a challenging task. Traditionally, short synthetic peptide sequences have been assembled on lysine-based multiple antigenic peptide (MAP) scaffolds by iterative coupling of amino acid residues employing solid phase peptide synthesis **[16,17]**. Our laboratory explored the utility of azide-alkyne click chemistry for displaying two or four copies of alkyne-labelled proteins on azide-terminated MA) scaffold **[18]**. Here, appropriately engineered recombinant proteins were labelled with alkyne groups using transpeptidase sortase and were subsequently clicked to the azide-terminated dendritic scaffold using copper-catalyzed azide-alkyne cycloaddition (CuAAC) reaction **[19,20]**. The ease of synthesis of per-6-azido-β-cyclodextrin and an opportunity to expand the valency to seven has inspired us to explore β-CD as a template for covalent display of peptides and large proteins. We have chosen two proteins, namely, PspA (pneumococcal surface protein A) and RrgB (a pilus protein), respectively, form *Streptococcus pneumoniae* with the long-term goal of exploring these proteins as multivalent vaccine candidates.

## Materials and methods

Fmoc-propargylglycine (Fmoc-D-Pra-OH) was purchased from Anaspec, USA. Fmoc-Gly and Wang resin was procured from Novabiochem, USA. 1-Hydroxybenzotriazole (HOBt) was purchased from GL Biochem, China. Oligonucleotide Primers were custom synthesized from Sigma-Aldrich, USA. PCR reagents, Taq Polymerase (Platinum HiFi) was obtained from Invitrogen, USA, Plasmid Miniprep kits, Gel extraction buffers, PCR purification kits, Ni-NTA beads, were obtained from Qaigen,USA. pET23b plasmid, T7 promoter /terminator primer was supplied by Novagen Inc., USA. All other chemicals and solvents used in the study were obtained from Sigma-Aldrich, USA.

### Synthesis of per-6-deoxy-6-azido-β-cyclodextrin [β-CD(N_3_)_7_] (1)

β-CD was converted to per-6-deoxy-6-azido-β-cyclodextrin in two steps following the procedure described by Ashton *et al* **[15]**. The primary hydroxyl groups of β-CD were replaced with iodine by treatment with Ph_3_P and I_2_ at 70°C in DMF. Subsequent reaction of per 6-deoxy-6-iodo-β-CD with NaN_3_ resulted in the nucleophilic replacement of iodine by azide group. The product (per 6-deoxy-6-azido-β-CD) was purified by RP-HPLC and characterized by mass spectrometry.

### Synthesis of Gly-Gly-D-Pra (2)

Gly-Gly-D-Pra was assembled on Wang resin by standard solid phase peptide synthesis (SPPS) protocols employing 9-Fluromethoxycarbonyl (Fmoc) chemistry using a peptide synthesizer (Applied Biosystems, 433A Peptide Synthesizer or Aaptec Endeavor 90-II). The synthesis was initiated by coupling Fmoc-D-Pra-OH (0.8 mmol) to the resin using DCCI/HOBt. This was followed by deprotection of Fmoc group and two coupling/deprotection cycles of Fmoc-Gly-OH (0.8 mmol each cycle) to complete the synthesis of **2**. The peptide was released from the resin by treatment with 95% TFA and precipitated in chilled diethyl ether. The crude material was extracted in water and purified by reverse-phase high performance liquid chromatography (RP-HPLC). ESMS: M+H, observed m/z 228.12 Da (theoretical 228.09 Da).

### Reverse-phase high performance liquid chromatography (RP-HPLC)

Analytical RP-HPLC was carried out using a C18 column (Phenomenex, Luna 5u, 4.6mm X 250mm). The peptide samples were eluted from the column by employing a linear gradient of solvent B (5–90% B in 130 min; solvent B, 80% acetonitrile in water containing 0.1% aqueous TFA; solvent A, 0.1% TFA; flow rate: 1 ml/min). Preparative RP-HPLC (LC-8A Shimadzu Preparative Liquid Chromatograph) was used for large-scale purification of peptides using a preparative column (Phenomenex preparatory C18 column, Luna 10 μ, 30 mm X 250 mm). The chromatography was carried out using the same solvent gradient system as above but at a flow rate of 30 ml/min.

### Cloning, expression and purification of recombinant proteins

Sortase A (SrtA) from *Staphylococcus aureus* bearing a C-terminal His_6_- tag was expressed and purified from *E*. *coli* as described previously **[21]**.

A 84-residues fragment of Pneumoccocal surface protein (PspA) from *Streptococcus pneumoniae* representing residues 203–286 appended with a LPNTG sortase recognition motif followed by a C-terminal His_6_- tag was expressed and purified from *E*. *coli* as described previously **[18]**. The purified protein was quantified by spectrophotometry using a molar extinction coefficient of 2560 M^-1^cm^-1^ at 280 nm.

RrgB(D1) plasmid DNA of *S*. *pneumoniae* encoding amino acid residues from 30 to 183 was amplified from genomic DNA using appropriate primers (Forward primer; 5’ CGCGCATATGGCTGGGACGACAACAACATCTGTTACC ‘3, Reverse primer;

5’GCGCCTCGAGACCGGTATTCGGAAGTCCTCCTCCTTTTGGATACACATGCGCATCCACAACATC’3) for incorporation of GGGLPNTG sequence followed by a His_6_ tag at the C- terminus. Fidelity of amplified PCR product and modified plasmid DNA (510 bp) encoding RrgB(D1) protein was established by DNA sequencing. The plasmid DNA isolated from the correct clone was transformed into *E*. *coli* BL21(DE3) for protein expression. The cells were grown in LB medium containing 100 μg/ml ampicillin at 37°C. At O.D._600_ ~ 0.6, protein expression was induced with 0.5 mM IPTG at 30°C for 6 hr. Cells were harvested by centrifugation (4000 rpm for 20 min at 4°C) and processed for Ni-NTA purification. The protein concentration was estimated by spectrophotometry using molar extinction coefficient of 12950 M^-1^cm^-1^ at 280 nm.

### Generation of alkyne-labelled proteins by sortase-mediated ligation

In a typical reaction, 0.5 mM of desired protein (PspA or RrgB) and peptide **2** (5 mM) were taken in an appropriate volume of buffer S (pH 7.5, 50mM Tris + 150mM NaCl + 5mM CaCl_2_ + 2 mM β-mercaptoethanol) and incubated at 37°C for 12 h in the presence of 20 μM SrtA. Subsequently, the reaction mixture was passed through a Ni-NTA column pre-equilibrated with buffer A (50 mM Tris + 150 mM NaCl, pH 7.5). The unbound material was collected and concentrated using a 15 ml Amicon concentrator (Cut-off = 3/10 KDa) at 4°C and subjected to size-exclusion chromatography for the removal of excess peptide **2**. An aliquot from the concentrated sample was analyzed by SDS-PAGE and mass spectrometry.

### Conjugation of peptide 2 to β-CD(N_3_)_7_ (1) by CuAAC reaction

Gly-Gly-D-Pra (0.7mM) was mixed with β-CD(N_3_)_7_ (0.1mM) in Buffer A. Click ligation was initiated by addition of freshly prepared sodium ascorbate (20 mM) and copper sulphate (10 mM) for 1 hr at room temperature. The reaction mixture was subjected to RP-HPLC and the product was analyzed by mass spectrometry.

### Conjugation of proteins to β-CD(N_3_)_7_ (1)

In general, click reaction between alkyne-labeled protein and β-CD(N_3_)_7_ was carried out in the same way in Buffer A as above with a 7-fold molar excess of labeled protein over **1**. PspA-alkyne/ RrgB (D1)-alkyne (0.7 mM) was incubated with **1** (0.1 mM) in the presence of sodium ascorbate (20 mM) and copper sulphate (10 mM) for 1 hr at room temperature to get the final protein dendrimer product. The conjugation reaction of RrgB-alkyne was carried out as above in buffer A containing 20% DMSO to enhance the solubility of the reactants. The reactions for both proteins were monitored by SDS-PAGE and size-exclusion chromatography. The yield of the reaction was calculated by comparing the area under the curve for reactant peptide and the dendrimer product.

### Size-exclusion chromatography

The separation of dendrimeric protein from the unreacted monomeric protein was carried out by FPLC on a Superdex 200 (10/300 GL) or Superdex 200 Increase 10/300 GL column using Buffer A at a flow rate of 0.5 ml/min. Appropriate fractions (0.5 ml each) were pooled, concentrated, and analyzed by SDS-PAGE.

## Results

### CuAAC -mediated ligation of Gly-Gly-D-Pra (2) to β-CD(N_3_)_7_ (1)

**Fig 1:**
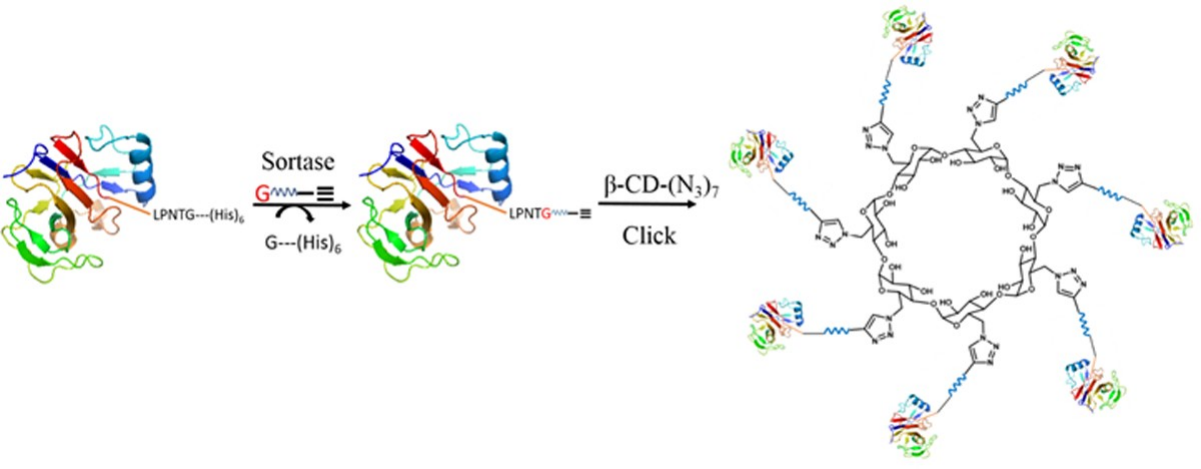
Schematic representation of Sortase—click strategy for the semisynthesis of multivalent protein dendrimers on a cyclodextrin scaffold. A Gly-terminated alkyne moiety can be installed at the C-terminus of any recombinant protein engineered with a LPNTG pentapeptide motif by sortase-mediated peptide ligation. The alkyne-labelled protein is then clicked with per-6-deoxy-6-azido-β-cyclodextrin [β-CD(N_3_)_7_] through Huisgen cycloaddition reaction.

Transpeptidase Sortase A efficiently catalyzes the ligation of LPXTG (X = any residue other than Pro) polypeptide to an aminoglycine terminated moiety. We have earlier applied this enzymatic approach for installing azide/alkyne orthogonal label on proteins for conjugating them to complementary lysine-based divalent or tetravalent multiple antigenic peptide (MAP) scaffolds using copper catalyzed azide-alkyne cycloaddition (CuAAC) click chemistry. The successful synthesis of divalent and tetravalent proteins prompted us to explore if the Sortase-Click strategy can be adapted to generate heptavalent display of large proteins on the β-CD scaffold (Fig 1).

**Fig 2:**
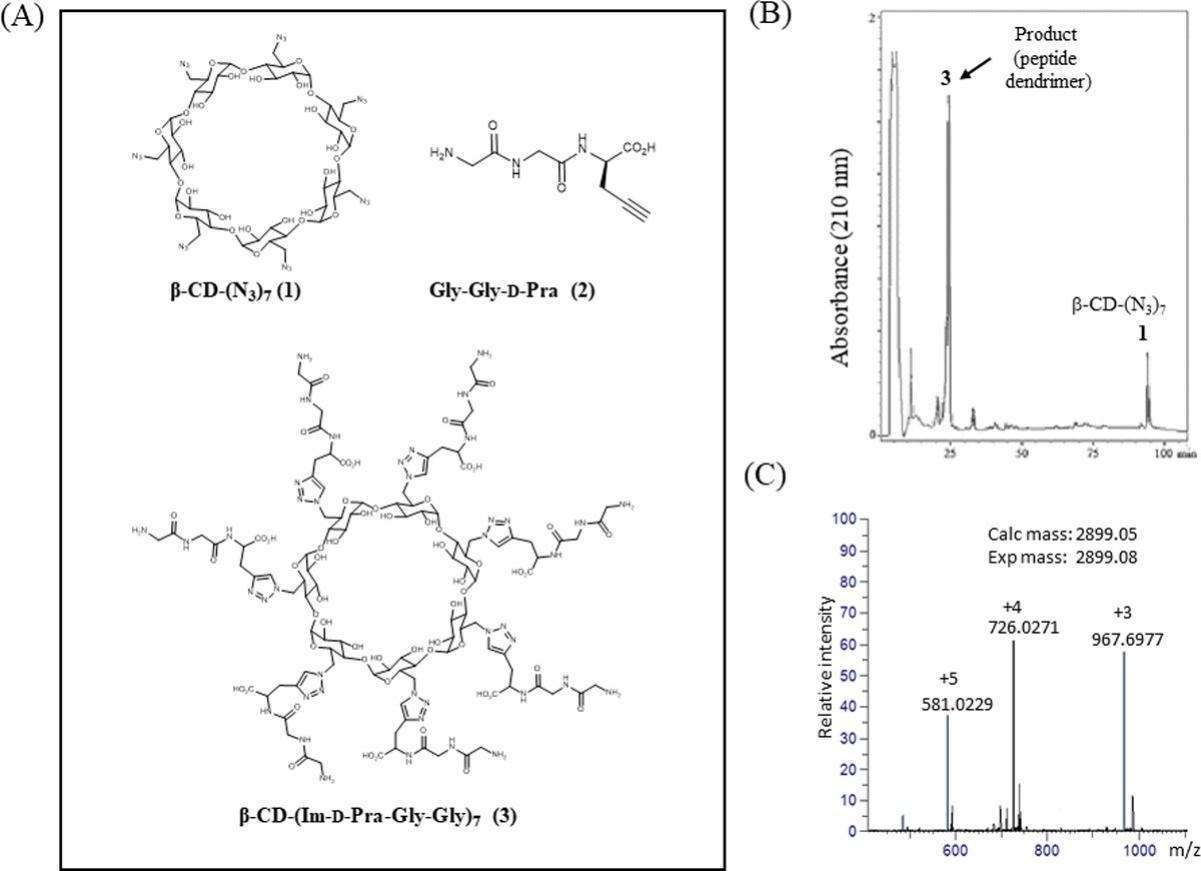
(A) Chemical structures used in the study: per-6-deoxy-6-azido-β-cyclodextrin (**1**, β-CD(N_3_)_7_), Gly-Gly-D-Pra (**2**) and click ligation product **3**, β-CD-(Im-D-Pra-Gly-Gly)_7_] of **1** and **2**. (B) RP-HPLC profile of the click reaction between **1** and **2**, (C) ESI-MS of the product peak (**3**). The calculated m/z value is indicated in parenthesis.

We first derivatized β-CD with azide functionalities to synthesize per-6-deoxy-6-azido-β-CD in two steps as described by Ashton *et al* **[15]**. The primary hydroxyl groups were replaced with iodine by treatment of β-CD with Ph_3_P and I_2_ at 70°C in DMF. Subsequently, per-6-deoxy-6-iodo-β-CD was converted to per-6-deoxy-6-azido-β-CD by nucleophilic replacement of iodine with azide group by treating with NaN_3._ The per- 6-deoxy-6-azido-β-CD (1, Fig 2A) was purified by RP-HPLC and MALDI-TOF of the purified [β-CD(N_3_)_7_] yielded two peaks, m/z 1332.26 Da and 1348.22 Da which fit well to the sodium and potassium adduct of per-6-deoxy-6-azido- β-CD, respectively (calculated mass, 1309.41 Da).

In order to gauge the efficiency of click ligation to the β-CD scaffold and assess the extent of site occupancy, we synthesized a tripeptide **2** with a propiolyl moiety (Fig 2). We tested the ability of **2** to react with β-CD(N_3_)_7_ (**1**). Interestingly, RP-HPLC profile of the CuAAC reaction, carried out for 1 h as described in Methods, showed the presence of only one major product peak (Fig 2B). ESI-MS analysis of the product peak (Fig 2C) yielded a mass of 2897.33 Da which fits, within experimental error, to an adduct of β-CD(N_3_)_7 _(1309.41 Da) and seven copies of the peptide **2** (7x227.09 = 1589.63) indicating facile formation of a heptavalent peptide dendrimer (calculated mass, 2899.04 Da).

### CuAAC -mediated ligation of PspA-alkyne to β-CD(N_3_)_7_ (1)

**Fig 3:**
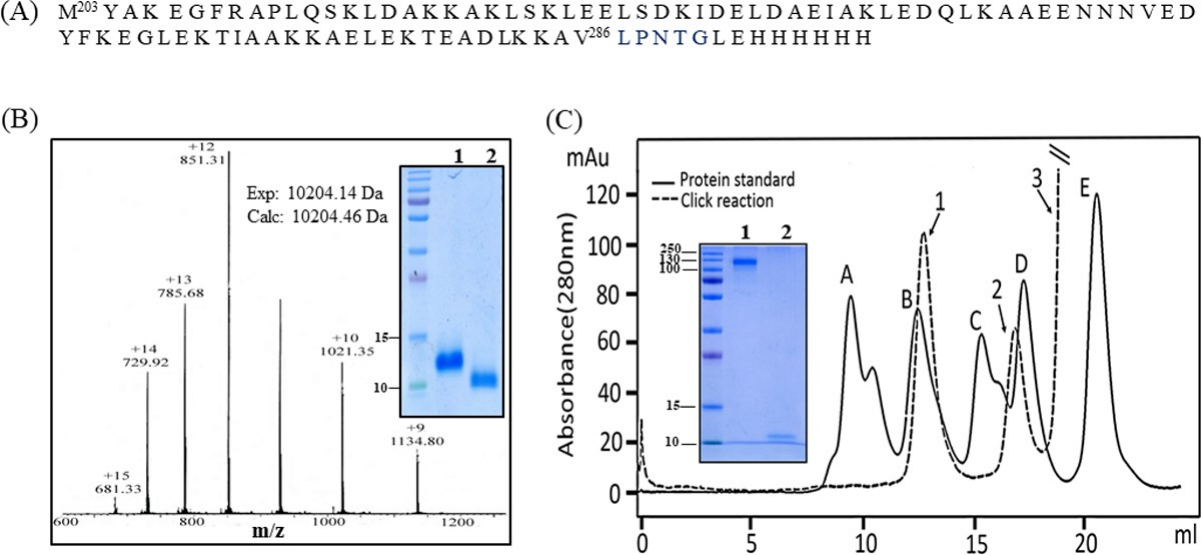
Purification, characterization and alkyne labeling of PspA construct. (A) Amino acid sequence of the expressed PspA^203-286^-LPNTG-His_6_, (B) ESI-MS of PspA-alkyne protein: The experimental mass of alkyne-labeled PspA was found to be 10204 Da which is in accord with the sortase-mediated installation of Gly-Gly-D-Pra (**2**) to the C-terminal of PspA construct. Inset shows the SDS-PAGE of the expressed PspA (lane 1) and PspA-alkyne (lane 2). (C) Semisynthesis of PspA dendrimer: CuAAC click ligation of PspA-alkyne and β-CD(N_3_)_7_ (**1**) was carried out at 37 C for 1 h and the reaction mixture was subjected to size-exclusion chromatography (shown with broken lines) using a Superdex 200 Increase 10/300 GL column. The individual peaks (dendrimer, peak1 and unreacted PspA-alkyne, peak 2) were pooled, concentrated and analyzed by SDS-PAGE. The last peak contains click reagents (sodium ascorbate, copper sulfate). Inset shows the SDS-PAGE profile of peak 1 (dendrimer) and peak 2 (PspA-alkyne). The chromatogram shown with solid line represents the profile of a standard sample comprising thyroglobulin (peak A, M.wt. 670000), γ-globulin (peak B, M.wt. 158000), ovalbumin (peak C, M.wt. 44000), myoglobin (peak D, 17000), and vitamin B12 (peak E, M.wt. 1350).

For exploring display of longer polypeptides on CD-scaffold, we chose a domain of PspA corresponding to residues 203–286 (Fig 3A). This domain was engineered to contain a LPNTG motif for recognition by Sortase A and a hexa-His tag at the C-terminus to facilitate purification of the expressed protein as well as separation of the alkyne-labelled protein from the reaction mixture after sortase-mediated ligation. The protein was expressed and purified as described earlier. SDS-PAGE analyses showed a single band migrating around 12 KDa (Fig 3B, inset, lane 1). ESI-MS analysis of purified protein showed a mass of 11117.05 Da that fits well with the theoretical mass of 11117.55 Da indicating high purity of the protein.

Next, we installed the alkyne group on PspA^203-286^-LPNTG-His_6_ (Fig 3A) by sortase-mediated ligation. The ligation of Gly-Gly-D-Pra (**2**) to the protein was carried out at pH 7.5 in the presence of 20 μM SrtA at 37°C for 12 h as described in Methods. The reaction mixture was passed through Ni-NTA affinity matrix for removing the unlabeled protein (His-tagged), SrtA (His-tagged), and the GLEH_6_ fragment from the reaction mixture. Analysis of the unbound material from the Ni-NTA matrix by SDS-PAGE showed the presence of a single band corresponding to PspA-alkyne (Fig 3B, inset, lane 2). ESI-MS analysis of purified protein showed an experimental mass of 10204 Da in accord with installation of **2** (Gly-Gly-D-Pra) at the C-terminus of PspA^203-286^ (Fig 3B).

The alkyne labelled PspA (PspA-alkyne) was conjugated to β-CD(N_3_)_7_ scaffold (1) in the presence of 10 mM copper sulfate and 20 mM sodium ascorbate for 1 hour at room temperature. The reaction mixture was subjected to size-exclusion chromatography, using a Superdex 200 Increase 10/300 GL column, for the separation of protein dendrimer from unreacted protein. A standard protein mixture sample, containing thyroglobulin (670000 Da), γ-globulin (158000 Da), ovalbumin (44000 Da), myoglobin (17000 Da), and vitamin B12 (1350 Da), was also chromatographed under similar conditions for comparative analyses. The profile of click ligation reaction (traces with broken lines, Fig 3C) showed two well-resolved protein peaks (peak 1 and peak 2) with an additional third peak (peak 3) of click reagents (sodium ascorbate, copper sulfate). The protein dendrimer (peak 1) eluted (12.74 ml) slightly later than γ-globulin (12.50 ml). Consistent with this, SDS-PAGE analysis (Fig 3C, inset), of the peak 1 showed a single band around 130 kDa. The peak 2 yielded a protein band corresponding to that of the unreacted PspA-alkyne. Taken together, these results show that PspA dendrimer (calculated mass ~ 72740 Da) migrated as much higher molecular weight species in both SDS-PAGE and size-exclusion chromatography. Notwithstanding this, absence of multiple protein bands in the SDS-PAGE profile of the peak 1 comprising the dendrimer suggests saturation of the heptavalency in CD. The yield of PspA dendrimer based on the peak area was estimated as 70% (Fig 3C).

### Covalent display of a domain of RrgB

**Fig 4:**
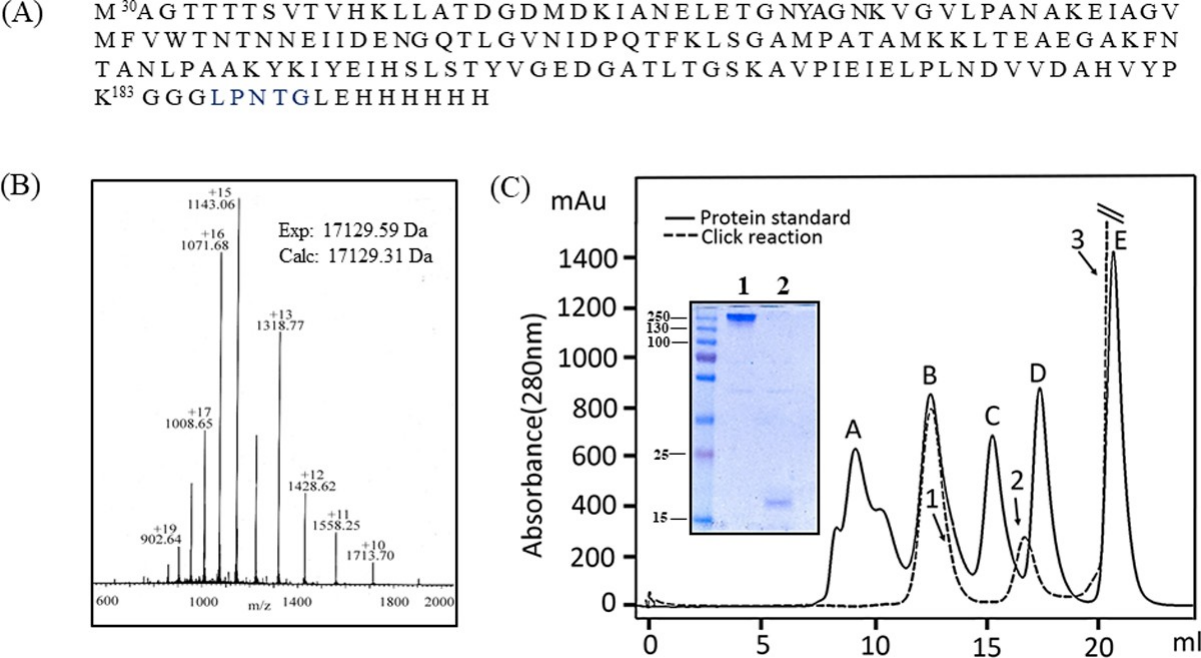
Characterization of RrgB-alkyne and semisynthesis of RrgB dendrimer. (A) Amino acid sequence of the expressed RrgB(D1)-LPNTG-His_6_, (B) ESI-MS of RrgB-alkyne: The experimental mass of RrgB-alkyne was observed as 17129 Da consistent with the sortase-mediated ligation of **2** on RrgB(D1)-LPNTG-His_6_. (C) Semisynthesis of RrgB dendrimer: CuAAC click ligation of RrgB-alkyne and β-CD(N_3_)_7_ (**1**) was carried out and processed by size-exclusion chromatography as described in Fig 3 (click reaction, broken lines; standard protein sample; solid lines). Inset shows SDS-PAGE of the individual chromatographic peaks (dendrimer, peak1 and unreacted RrgB-alkyne, peak 2). The last peak comprises of click reagents.

In order to generalize the sortase-click strategy for the display of large proteins on β-CD scaffold, we cloned and expressed a domain D1, comprising residues 30–183 of pilus forming RrgB protein from *Streptococcus pneumoniae* (Fig 4A). The protein was expressed nested with a “LPNTG” sortase recognition motif followed by a hexa-histidine tag (RrgB(D1)-LPNTG-His_6._). ESMS analysis generated a mass of 18042.32 Da in agreement with its calculated mass (18042.28 Da). With a highly purified preparation at hand, we proceeded to the alkyne labeling of the RrgB(D1) protein. The labeling reaction of RrgB with **2** was carried out in much the same way as that of PspA. After Ni-NTA chromatography, the purified material produced a single band on SDS-PAGE and yielded a mass of 17129.59 Da by ESMS (Fig 4B) in close accord (without N-terminal methionine residue) with the installation of **2** at the C-terminus of RrgB(D1). The mass of RrgB(D1)-alkyne was consistent with the calculated value (17129.31 Da) indicating the high purity of the alkyne-labeled protein.

Next, we carried out the click conjugation of RrgB(D1)-alkyne to β-CD(N_3_)_7_ (1) scaffold in a similar fashion as described above for the PspA-alkyne and processed the reaction mixture through size-exclusion chromatography using a Superdex 200 10/300 GL column. Interestingly, the chromatographic profile (Fig 4C) showed a pattern similar to that observed in the PspA conjugation reaction. The profile displayed three peaks corresponding to the high molecular weight dendrimer (peak 1), unreacted RrgB(D1)-alkyne (peak 2), and click reagents (peak 3) respectively. The dendrimer peak1 eluted at the same position as γ-globulin (158000 Da) and migrated as a considerably higher molecular weight species (250 kDa, calculated 122 kDa) on SDS-PAGE (Fig 4C, inset, lane 1). This abnormal behavior of both protein dendrimers (PspA and RrgB) on size-exclusion chromatography and SDS-PAGE may be a consequence of alteration in hydrodynamic volume and/or SDS binding due to unique spatial organization afforded by β-CD. It is pertinent to mention here that the size exclusion columns are traditionally calibrated with standard protein samples assuming a correlation between elution volume and hydrodynamic volume or radius of gyration. However, this may not be true for all polymers since they come in different shapes (linear, branched, star etc.) [22].

## Discussion

Assembly of chemically well-defined protein dendrimers by purely chemical methods is limited by the sequence length and associated purification complexities. These limitations have been partly overcome by advances in peptide fragment condensation and click chemistry approaches. However, the lack of a general route for assembly of a dendrimer composed of large proteins remains a formidable problem. This is further underscored by the description of limited examples of defined protein dendrimers in literature. Some examples include a trivalent antigen binding construct built on a trialdehyde scaffold using an aminoxy derivatized Fab fragment **[23]**, divalent and tetravalent dendrimers of GFP or collagen binding protein CNA35 by NCL using branched peptides terminating with N-terminus Cys residues and respective thioester derivatized proteins **[24]**. A combinatorial approach of native chemical ligation (NCL) and CuAAC was also explored with a tripropargylamine scaffold for the assembly of trivalent protein G dendrimers **[25].** Recently, we exploited the peptide ligation propensity of transpeptidase sortase for installing orthogonal groups compatible with click chemistry for synthesis of chemically defined protein dendrimers **[18]**. A similar two-step approach using a combination of sortase-mediated labelling and click chemistry was earlier employed for site-specific modification of recombinant antibodies **[26]**.

Cyclodextrin (CD) based multivalent scaffold comprises one of the most easily accessible and versatile scaffolds for multiple display of ligands because of its spatial conformation afforded by limited flexibility of the cyclic core. CD scaffold is especially attractive for biotechnological and clinical applications due to its non-immunogenic nature and low intrinsic pharmacological activity. These attributes together with high degree (heptavalent) of first generation branching make CD scaffolds ideal for the covalent display of peptides and proteins in a variety of clinical and mechanistic settings. The specific conjugation of peptides and proteins to CD requires installation of appropriate orthogonal functionality on both, CD and protein. While derivatization of CD is easily accessible, chemically defined protein- CD conjugation is limited by readily available orthogonal protein labelling approaches. In recent years, however, sortase-mediated peptide ligation reaction has emerged as a robust tool for installing orthogonal functionality at the C-terminal of proteins. The ease of synthesis of β-CD(N_3_)_7_ as well as its commercial availability together with facile alkyne-labelling of proteins by sortase-mediated ligation has been our major inspiration for using the Sortase-Click strategy for the construction of multivalent CD—protein conjugates.

We first tested the click ligation efficiency of a short tripepetide **2** (Gly-Gly-D-Pra), equipped with an alkyne group, to β-CD(N_3_)_7_ (**1**) and observed complete occupancy of valency with predominant formation of a heptavalent peptide dendrimer. Interestingly, the click reaction of labelled proteins also proceeded in a similar fashion as that of the peptide and produced fully occupied heptavalent dendrimers as judged by size-exclusion chromatography and SDS-PAGE electrophoresis. The absence of dendrimers with intermediate valency (hexavalent or lower) with the short peptide as well as large proteins is a striking result that suggests a favorable disposition of azido groups in β-CD(N_3_)_7_ for click reaction.

Given the ease with which recombinant proteins carrying a pentapeptide motif at their carboxy terminus can be appended with an alkyne group and commercial availability of CD, Sortase-click strategy may be considered a generic approach for the biomolecular assembly of peptide/protein ligand on a cyclodextrin scaffold. The Sortase-click approach described here offers rich possibilities in macromolecular assemblage of a variety of peptides/proteins on CD scaffold for biotechnological and medical applications. In this context, it would be interesting to explore conjugation of two or more proteins on the same azido-CD scaffold. This may be simply achieved through the control of protein stoichiometry during click conjugation provided product isolation does not entail complex purification protocols.
